# Accelerated-Aging Screening Data for Polymer Liners in Oil and Gas Flexible Composite Pipes: A Communication

**DOI:** 10.3390/polym18121524

**Published:** 2026-06-18

**Authors:** Pingyuan Xia, Tianyi Ma, Lin Lei, Qingxia Wang, Xiaomin Lu, Xiaolin Zhu, Yan Yan, Jiaqiao Zhang

**Affiliations:** 1 Jiangsu Zhengdao Ocean Technology Co., Ltd., Nantong 226000, China; xiapingyuan@ztoc.com.cn (P.X.); alicia.wang@ztoc.com.cn (Q.W.); luxiaomin@ztoc.com.cn (X.L.); zhuxiaolin@ztoc.com.cn (X.Z.); 2 School of Mechanical Engineering, Southeast University, Nanjing 211189, China; tianyima99@163.com (T.M.); 220250339@seu.edu.cn (L.L.); 101012319@seu.edu.cn (Y.Y.)

**Keywords:** oil and gas transportation, flexible composite pipe, polymer liner, accelerated aging, hydrocarbon swelling

## Abstract

This Communication reports limited engineering screening data on polymer liner candidates for flexible composite pipes used in oil and gas service. Three exposure conditions were considered: hydrothermal aging in superheated water, thermal-oxidative aging in dry air, and hydrocarbon-medium exposure. Superheated-water immersion for up to 1000 h, dry-air aging for 168 h, and 7-day hydrocarbon exposure were used to describe changes in tensile properties, Shore hardness, mass, and thickness. Complete replicate records were available only for the thermal-oxidative aging dataset; therefore, most hydrothermal and hydrocarbon-medium results are reported as descriptive summary data. In the recorded data, EPDM formulation CL-2-1 retained approximately 89% of its tensile strength after 1000 h in superheated water. Sample L showed a smaller mean tensile-strength decrease than Sample Z after 168 h at 150 °C in dry air. In the hydrocarbon-medium summary data, XL95A/05B-S1 showed lower mass increase and smaller tensile-strength and yield-stress decreases than PERT XRT70H across the tested temperature range. The Communication provides case-specific screening evidence and identifies the need for replicated testing, statistical analysis, longer aging series, and structural characterization before general material-selection or durability conclusions are made.

## 1. Introduction

Global oil and gas exploration are expanding toward extreme environments, continuously raising pipeline operating temperatures. In deep terrestrial and deepwater offshore oil and gas fields, produced fluids are transported at stable temperatures of 100–150 °C, with some high-temperature wells exceeding 150 °C [1]. These extreme conditions pose accelerated corrosion challenges for traditional steel pipelines, driving the adoption of flexible composite pipes as the preferred solution for deep well transportation. Flexible composite pipes offer superior corrosion resistance, excellent coilability, and convenient installation characteristics, with advantages particularly pronounced in deepwater environments [2,3]. However, the ultimate reliability of these pipes depends on the performance of the polymer liner [3]. When the liner fails, the entire pipe loses its service value [4].

Existing studies on polymer liner evaluation have mainly progressed along the following directions. In terms of standards and single operating-condition evaluation, international standards (e.g., ISO 9080:2012 [5] on long-term hydrostatic strength evaluation and ASTM D573 on test methods for rubber thermal aging) provide a methodological basis for performance assessment under a single operating condition.

In fundamental research on failure mechanisms, for hydrolytic degradation, researchers such as Krauklis and Starkova [6] systematically reviewed modeling methods and durability prediction approaches for polymers and polymer composites under environmental aging; Khalid et al. [7] provided a detailed review of damage mechanisms in polymer liners used in oil and gas pipelines caused by gas and liquid permeation, with particular emphasis on the hydrolysis sensitivity of PA in high-temperature water environments.

Regarding thermal-oxidative degradation, Frigione and Rodríguez-Prieto [8] elaborated on the application and limitations of accelerated aging procedures in predicting long-term polymer performance; Zhou et al. [9] systematically investigated the thermal aging behavior of EPDM elastomers at different temperatures and proposed a quantitative evaluation method for thermal-aging failure. In addition, for long-term life prediction, the application of the Arrhenius model has been validated in multiple studies: Maxwell and Broughton [10] presented a classic review of accelerated aging methods and polymer life prediction techniques; Starkova et al. [11] further discussed multiscale modeling and durability prediction methods for polymers and polymer composites under environmental aging; and Plota and Masek [12] systematically summarized life prediction methods for degrading polymers, including the Arrhenius model and the time–temperature superposition principle (TTSP). Recent studies, such as the research by Aniśko-Michalak et al. [13] on the long-term thermal-oxidative aging of polyethylene, and the experimental work by Doan et al. [14] on long-term creep of fiber-reinforced thermoplastic polymer pipes under high stress, have further supplemented the understanding of polymer property evolution under service conditions.

For polymer liners used in oil and gas flexible composite pipes, material degradation is strongly dependent on the transported medium and service environment [15]. In high-temperature water-injection pipelines, hydrolytic degradation may occur due to the interaction between water molecules and hydrolysis-sensitive chemical bonds. In dry gas pipelines, thermal-oxidative aging is mainly driven by the combined effects of heat and oxygen [16,17]. In crude-oil transportation, hydrocarbon absorption and solvent swelling may reduce dimensional stability and mechanical properties [18,19,20]. Therefore, these three representative degradation risks are directly related to the practical service scenarios considered in this study and provide the basis for the following experimental design.

However, while these studies have made progress within specific operating conditions or failure-mechanism frameworks, they generally lack a systematic consideration of complex operating conditions and the specificity of modified materials. In particular, when confronted with complex realities in oil and gas transportation, such as hydrolysis threats in water injection pipelines, thermal-oxidative degradation in dry gas pipelines, and swelling-induced damage in crude oil pipelines, engineers still lack a scientific evaluation framework that systematically links operating condition, failure mechanism, and material selection, resulting in material selection that remains largely dependent on empirical judgment and lacks theoretical support.

Based on these observations, this Communication reports and organizes available accelerated-aging screening data for polymer liners used in flexible composite pipes. The work is presented as a limited engineering dataset rather than as a fully statistically validated universal material-selection study. The literature-informed framework links three service scenarios with corresponding evaluation risks: hydrothermal aging for water-injection-related exposure, thermal-oxidative aging for dry-gas-related exposure, and hydrocarbon uptake and swelling for crude-oil-related exposure. The scope is limited to the measured mechanical, hardness, mass, and thickness changes available in the present datasets. The main contribution is to present case-specific screening observations and to identify the additional replicate, statistical, long-term, and structural data required before broader material-selection or durability conclusions can be made.

## 2. Materials and Methods

### 2.1. Experimental Design and Accelerated Aging Methods

For the purposes of this Communication, three service-related risks were compared using the available accelerated-aging tests. The selected exposure conditions generated descriptive screening data for material applicability across hydrothermal, thermal-oxidative, and hydrocarbon-medium risks.

For hydrothermal aging assessment, EPDM rubber samples with approximately 2 mm initial thickness were placed in sealed high-temperature, high-pressure reactors filled with superheated water at 150 °C. Samples were removed at 0, 200, 368, 548, 840, and 1000 h, cooled to room temperature, and tested.

For thermal-oxidative aging assessment, modified polyolefin samples were placed in a temperature-controlled oven at 150 °C for 168 h. Tensile strength, yield stress, and elongation at break were measured before and after aging. For Sample Z and Sample L, three individual specimen values before aging and three individual specimen values after aging were available; these data were therefore summarized as mean ± standard deviation (SD).

For hydrocarbon-medium immersion assessment, the simulated medium consisted of 70 vol% kerosene and 30 vol% nitrogen, with total pressure maintained at 5.5 MPa. Seven-day experiments were conducted at 75 °C, 85 °C, and 95 °C. Thickness, mass, tensile strength, yield stress, and elongation at break were compared before and after 7-day hydrocarbon-medium exposure. The results are reported as experimental summary percentage changes based on the recorded measurements. The kerosene and nitrogen used in the simulated medium were supplied by Jiangsu Zhengdao Ocean Technology Co., Ltd. (Nantong, China).

### 2.2. Materials and Characterization

For hydrolytic degradation assessment, two EPDM formulations were evaluated. The L-1 formulation used Arkema EPDM K6160D (Arkema, Colombes, France) blended with EPM K3050 (ARLANXEO, Maastricht, The Netherlands) and peroxide vulcanization for wrap-gap curing. The CL-2-1 formulation used EPDM K6160D alone with semi-effective vulcanization and was designed for single-pass co-extrusion. These formulations reflect a practical trade-off in rubber compounding: the binary ethylene propylene monomer (EPM) contains only saturated C-C bonds and lacks third monomer units, which is associated with higher thermal stability but poor processing characteristics [21,22,23].

For thermal-oxidative stability assessment, two formulations of modified polyolefin XL95A/05B, labeled Sample Z and Sample L, were evaluated. The present measurements were used to compare short-term property retention under the specified aging condition.

For swelling resistance assessment, three materials were compared. PERT XRT70H (TotalEnergies Petrochemicals & Refining S.A./N.V., Polymers Division, Brussels, Belgium) [24,25] served as a reference thermoplastic polyolefin, and XL95A/05B-S1 and XL95A/05B-S (Lianqiao New Materials Science and Technology Co., Ltd., Weihai, China) represented two modified polyolefin formulations. The comparison focused on measured mass, thickness, and mechanical-property changes under the selected hydrocarbon-medium immersion conditions.

Tensile properties, including tensile strength, yield stress, and elongation at break, were measured using dumbbell-shaped specimens according to the relevant polymer tensile testing procedure. Shore hardness was measured using a Shore A hardness tester. For swelling evaluation, specimen mass and thickness were measured before and after immersion. Before post-immersion measurement, residual surface medium was gently removed from the specimen surface. The mass change rate and thickness change rate were calculated relative to the initial values before immersion.

Data reporting followed the available experimental records. For the thermal-oxidative aging dataset, three individual specimen values were available for each material before and after aging; these data were summarized as mean ± standard deviation (SD). Because complete individual replicate records were not available for the hydrothermal-aging and hydrocarbon-medium datasets, those results are reported only as experimental time-point or percentage-change summary values. No inferential statistical analysis was performed for these summary datasets, and they were not used to support statistical significance claims.

Tensile properties were measured using a CMT5105 universal testing machine (MTS Industrial Systems (China) Co., Ltd., Shanghai, China), and Shore hardness was recorded using a LX-A Shore A durometer (Shanghai Sanliang Tools Co., Ltd., Shanghai, China). Specimen mass was measured using an ME104E/02 analytical/electronic balance (Mettler-Toledo Instruments (Shanghai) Co., Ltd., Changzhou, China), and thickness was measured using a ZB-101 thickness gauge (Jiangsu Zhengrui Taibang Electronic Technology Co., Ltd., Yangzhou, China). Thermal-oxidative aging was conducted in a DHG-9145A temperature-controlled oven (Shanghai Yiheng Scientific Instrument Co., Ltd., Shanghai, China). Hydrothermal aging and hydrocarbon-medium exposure were conducted in 10 L sealed high-temperature high-pressure reactors/autoclaves equipped with nitrogen pressure control (Weihai Yixin Chemical Machinery Co., Ltd., Weihai, China).

## 3. Experimental Results

### 3.1. Hydrolytic Resistance of EPDM Rubber

**Figure 1:**
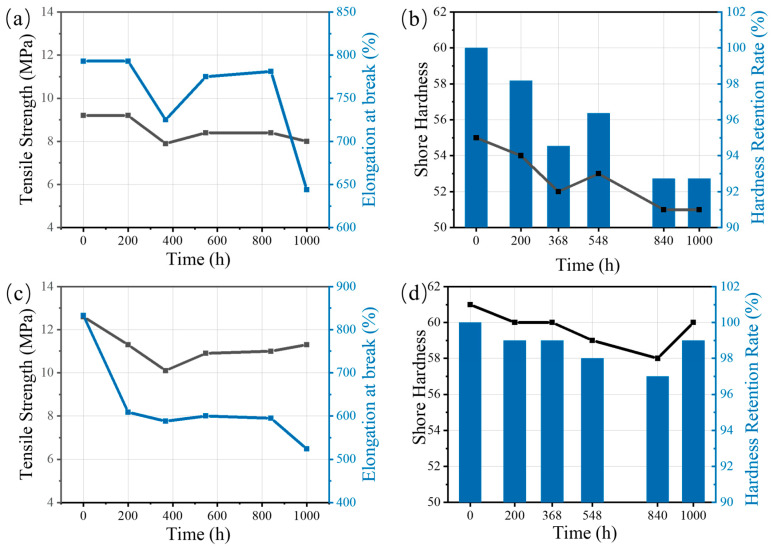
(**a**) Tensile strength of L-1 compound in superheated water at 150 °C. (**b**) Shore hardness of L-1 rubber compound in superheated water at 150 °C. (**c**) Tensile strength of CL-2-1 compound in superheated water at 150 °C. (**d**) Shore hardness of CL-2-1 rubber compound in superheated water at 150 °C. Values are shown as available summary data; no error bars are shown because complete replicate records were not available for statistical analysis.

Figure 1a,b show the tensile strength and Shore hardness of L-1 after treatment in superheated water at 150 °C. In the recorded experimental data, the tensile strength values at initial, 200 h, 368 h, 548 h, 840 h, and 1000 h were 9.2, 9.2, 7.9, 8.4, 8.4, and 8.0 MPa, respectively. The corresponding elongation values were 793, 793, 725, 775, 781, and 644%, and the Shore A hardness values were 55, 54, 52, 53, 51, and 51. The intermediate fluctuation is described only as an observed trend in the recorded dataset. Because complete replicate values were not available for this dataset, Figure 1 should be interpreted as a descriptive summary data rather than statistically analyzed data.

As shown in Figure 1c,d, CL-2-1 retained approximately 89% of its initial tensile strength after 1000 h of superheated-water exposure at 150 °C (12.6 MPa initially and 11.3 MPa after 1000 h in the recorded experimental data). Across initial, 200 h, 368 h, 548 h, 840 h, and 1000 h timepoints, the tensile strength values were 12.6, 11.3, 10.1, 10.9, 11.0, and 11.3 MPa, respectively. Elongation at break decreased from 833% to 524% over the same exposure period, and Shore A hardness changed from 61 to 60. Compared with L-1, CL-2-1 showed slightly higher tensile-strength retention but a larger decrease in elongation at break. Because complete replicate records were unavailable, this comparison should be regarded as descriptive rather than statistically validated.

Together, the two EPDM formulations retained approximately 87–89% tensile strength after 1000 h under the tested hydrothermal condition. These values indicate the recorded property changes under the selected accelerated-aging condition, but they do not establish statistically significant differences between the two formulations. Repeated measurements and structural characterization would be required before drawing stronger mechanistic or selection conclusions.

### 3.2. Thermal-Oxidative Stability Screening of Modified Polyolefins

The thermal-oxidative aging dataset, for which replicate values were available, is summarized as mean ± SD in Table 1. For Sample Z, tensile strength changed from 20.44 ± 0.02 MPa to 19.22 ± 0.21 MPa after 168 h at 150 °C, corresponding to a 5.97% decrease based on the mean values. For Sample L, tensile strength changed from 16.03 ± 0.24 MPa to 15.45 ± 0.72 MPa, corresponding to a 3.62% decrease. These results indicate limited short-term tensile-strength change under the tested dry-air aging condition.

The elongation at break of Sample Z increased from 189.13 ± 59.62% to 347.25 ± 24.78%. The large initial scatter in Sample Z elongation (coefficient of variation approximately 31.5%) means that this change should be interpreted cautiously. Sample L showed a smaller elongation change, from 279.05 ± 38.00% to 292.61 ± 41.54%. Because only one aging duration was available, these descriptive statistics were used for screening comparison and were not extended to long-term durability claims.

With one aging duration available for Sample Z and Sample L, the 168 h results are treated as short-term screening data for comparing property retention under the specified dry-air aging condition. No service-life prediction or statistically validated ranking was derived from this limited dataset.

Long-term thermal-oxidative behavior may involve antioxidant depletion, physical aging, creep effects, and changes in degradation pathway [26,27]. Longer aging series with repeated measurements would be needed before lifetime prediction could be attempted [28].

### 3.3. Hydrocarbon Swelling Resistance of Modified Polyolefins

**Figure 2:**
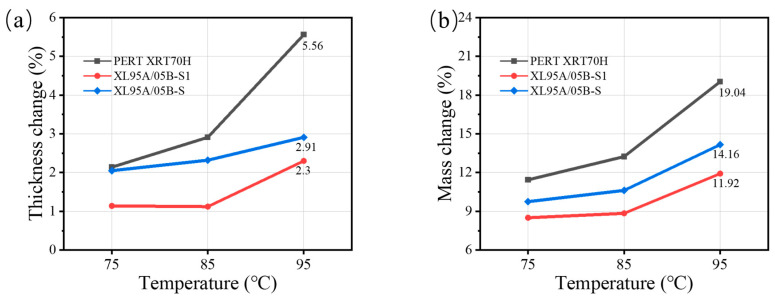
(**a**) Thickness variation rate of PERT XRT70H, XL95A/05B-S1, and XL95A/05B-S. (**b**) Mass change rate of PERT XRT70H, XL95A/05B-S1, and XL95A/05B-S. Values are experimental summary percentage changes; no inferential statistical analysis was performed because complete replicate records were not available.

**Figure 3:**
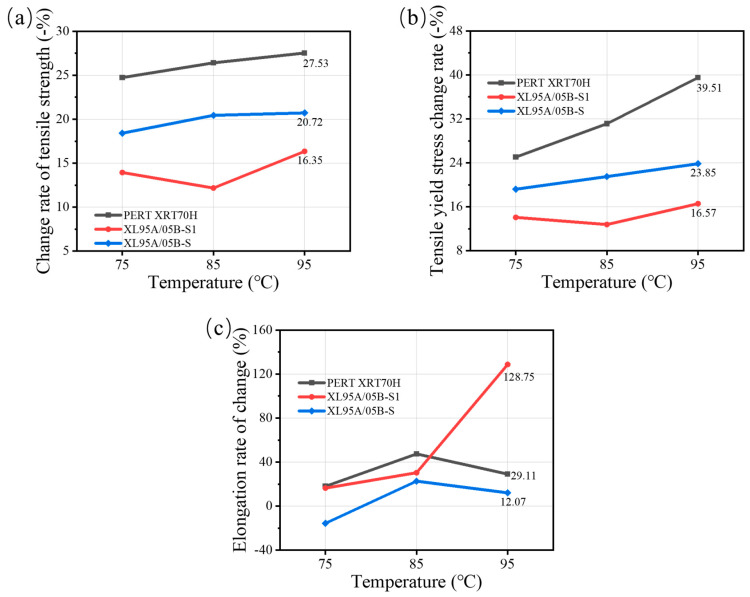
(**a**) Rate of change in tensile strength of PERT XRT70H, XL95A/05B-S1, and XL95A/05B-S. (**b**) Yield strength variation rate of PERT XRT70H, XL95A/05B-S1, and XL95A/05B-S. (**c**) Elongation change rate of PERT XRT70H, XL95A/05B-S1, and XL95A/05B-S. Values are experimental summary percentage changes; no error bars are shown because complete replicate records were not available.

Compatibility tests were conducted on PERT XRT70H, XL95A/05B-S1, and XL95A/05B-S. Before and after exposure, specimen mass and thickness were measured and variation rates were calculated relative to the initial values. Residual surface medium was gently removed before post-exposure measurement. Thickness and mass variations are shown in Figure 2, and mechanical-property changes are shown in Figure 3. The experimental dataset covered 7-day exposure at 75 °C, 85 °C, and 95 °C under 5.5 MPa in 70 vol% kerosene and 30 vol% nitrogen. Because complete replicate records were not available for this dataset, the values in Figure 2 and Figure 3, and Table 2 are reported as summary percentage changes and were used only for descriptive comparison under the tested conditions.

At 95 °C, XL95A/05B-S1 showed a 2.30% thickness increase, 11.92% mass increase, 16.35% tensile-strength decrease, and 16.57% yield-stress decrease. PERT XRT70H showed larger corresponding changes, including a 5.56% thickness increase, 19.04% mass increase, 27.53% tensile-strength decrease, and 39.51% yield-stress decrease. These summary values describe better dimensional and mechanical-property retention for XL95A/05B-S1 in this specific exposure condition, but the comparison was not treated as a statistically validated material ranking.

Across the 75–95 °C exposure range, XL95A/05B-S generally showed intermediate summary changes between PERT XRT70H and XL95A/05B-S1. At 95 °C, XL95A/05B-S showed a 14.16% mass increase, 20.72% tensile-strength decrease, and 23.85% yield-stress decrease.

Among the three tested materials, XL95A/05B-S1 showed the lowest mass increase at each tested temperature and smaller decreases in tensile strength and yield stress than PERT XRT70H in the available summary percentage data. This comparison provides descriptive screening evidence of relative swelling resistance under the tested hydrocarbon-medium conditions.

## 4. Scope and Limitations of the Communication

### 4.1. Evidence Boundaries of the Communication Dataset

The available results should be interpreted within the evidence boundaries of a Communication-format dataset. The study records changes in tensile properties, Shore hardness, mass, and thickness after selected accelerated-aging exposures. These measurements can be used to organize descriptive screening observations, but they do not establish molecular degradation mechanisms, statistically significant rankings for all datasets, or direct long-term service behavior.

Accordingly, the present analysis is limited to describing which measured indicators changed under each exposure condition. Strong engineering recommendations, service-life predictions, and mechanistic interpretations were avoided because complete replicate records, longer aging series, combined-medium tests, and direct structural characterization were not available for all datasets.

### 4.2. Limitations of Long-Term Durability Inference

In the present manuscript, the accelerated-aging data are not used to estimate actual long-term durability under real service conditions. The available 150 °C data only describe property changes under the tested laboratory exposure conditions.

Reliable durability assessment for polymer liners would require complete replicated datasets, multiple aging durations and temperatures, validation against long-term exposure, and direct structural characterization. Without these data, accelerated-aging results should be treated as screening observations rather than evidence for quantitative lifetime prediction.

### 4.3. Summary of Observed Responses and Evidence Boundaries

The available observations are summarized in Table 3 together with the corresponding evidence boundary for each tested exposure condition. Table 3 is intended as a dataset summary, not as a decision matrix or engineering material-selection rule.

For the water-injection-related exposure represented by 150 °C superheated water, the recorded summary data showed approximately 89.7% tensile-strength retention for CL-2-1 after 1000 h, compared with approximately 87.0% for L-1. Because complete replicate records were not available, this comparison should be interpreted descriptively.

For the dry-gas-related exposure represented by 150 °C dry-air aging for 168 h, Sample L showed a smaller mean tensile-strength decrease than Sample Z (3.62% versus 5.97%) in the available triplicate dataset. This result supports only short-term screening comparison under the tested condition.

For the crude-oil-related exposure represented by 7-day hydrocarbon-medium exposure, XL95A/05B-S1 showed lower mass increase and smaller tensile-strength and yield-stress decreases than PERT XRT70H across the tested temperature range in the available summary percentage data. Complete replicate testing would be required before statistical reliability could be assessed.

Combined-medium testing would be required before these screening observations are extended to mixed or multiphase service.

## 5. Conclusions

(1) Under 150 °C superheated-water exposure, L-1 and CL-2-1 retained approximately 87% and 89% of their initial tensile strength after 1000 h, respectively; however, these values are summary data without complete replicate records.
(2) For the 150 °C dry-air aging dataset, the available triplicate measurements showed limited mean tensile-strength decreases after 168 h: 5.97% for Sample Z and 3.62% for Sample L. The large scatter in elongation, especially for Sample Z, requires cautious interpretation.
(3) In the 7-day hydrocarbon-medium exposure tests at 75–95 °C, XL95A/05B-S1 showed lower mass increase and smaller tensile-strength/yield-stress decreases than PERT XRT70H in the available summary percentage data.
(4) The present results provide descriptive screening evidence only. Reliable material selection, degradation-mechanism identification, and service-life prediction require complete replicated testing, statistical analysis, longer aging series, combined-medium exposure, and direct structural characterization.

## Figures and Tables

**Table 1:** Mechanical properties of modified polyolefins (Sample Z and Sample L) before and after 168 h of thermal-oxidative aging at 150 °C. Values are mean ± SD (*n* = 3).

Material	Property	Initial Value (Mean ± SD)	Aged Value (168 h, Mean ± SD)	Rate of Change (%)
Sample Z	Tensile Strength (MPa)	20.44 ± 0.02	19.22 ± 0.21	−5.97
	Yield Stress (MPa)	20.41 ± 0.02	19.11 ± 0.12	−6.37
	Elongation at Break (%)	189.13 ± 59.62	347.25 ± 24.78	+83.61
Sample L	Tensile Strength (MPa)	16.03 ± 0.24	15.45 ± 0.72	−3.62
	Yield Stress (MPa)	15.22 ± 0.01	14.13 ± 0.13	−7.16
	Elongation at Break (%)	279.05 ± 38.00	292.61 ± 41.54	+4.86

**Table 2:** Summary swelling and mechanical-property changes of PERT XRT70H, XL95A/05B-S1, and XL95A/05B-S after 7-day hydrocarbon-medium exposure. Values are experimental summary percentage changes; no inferential statistics are reported for this summary dataset.

Material	Temperature	Dimensional/Mass Change (%)	Strength/Yield-Stress Change (%)	Elongation Change (%)
PERT XRT70H	75 °C	Thickness +2.14; mass +11.44	Tensile strength −24.74; yield stress −25.05	+18.03
PERT XRT70H	85 °C	Thickness +2.91; mass +13.24	Tensile strength −26.41; yield stress −31.12	+47.43
PERT XRT70H	95 °C	Thickness +5.56; mass +19.04	Tensile strength −27.53; yield stress −39.51	+29.11
XL95A/05B-S	75 °C	Thickness +2.05; mass +9.75	Tensile strength −18.41; yield stress −19.21	−15.63
XL95A/05B-S	85 °C	Thickness +2.32; mass +10.62	Tensile strength −20.44; yield stress −21.50	+22.68
XL95A/05B-S	95 °C	Thickness +2.91; mass +14.16	Tensile strength −20.72; yield stress −23.85	+12.07
XL95A/05B-S1	75 °C	Thickness +1.14; mass +8.50	Tensile strength −13.94; yield stress −14.08	+16.39
XL95A/05B-S1	85 °C	Thickness +1.12; mass +8.84	Tensile strength −12.17; yield stress −12.78	+30.36
XL95A/05B-S1	95 °C	Thickness +2.30; mass +11.92	Tensile strength −16.35; yield stress −16.57	+128.75

**Table 3:** Summary of observed material responses and evidence boundaries under the tested accelerated-aging conditions.

Service Scenario Represented	Evaluation Risk	Test Condition	Materials Compared	Observed Summary Result	Main Limitation
Water-injection-related exposure	Hydrothermal aging	150 °C superheated water, 1000 h	EPDM L-1; EPDM CL-2-1	L-1: ~87% tensile-strength retention; CL-2-1: ~89% tensile-strength retention with lower elongation retention.	Time-point summary data; no complete replicate records for statistical testing.
Dry-gas-related exposure	Thermal-oxidative aging	150 °C dry air, 168 h	Sample Z; Sample L	Sample Z: −5.97% mean tensile strength; Sample L: −3.62% mean tensile strength.	n = 3 before and after aging; single aging duration.
Crude-oil-related exposure	Hydrocarbon swelling	75–95 °C, 7 days; 70 vol% kerosene/30 vol% N2; 5.5 MPa	PERT XRT70H; XL95A/05B-S; XL95A/05B-S1	XL95A/05B-S1 showed the lowest mass increase and smaller tensile-strength/yield-stress decreases than PERT XRT70H across the tested temperature range.	Summary percentage changes; no complete replicate records for statistical testing.
Multiphase exposure	Combined exposure	Not tested	Not assessed	Not assessed	Requires combined-medium testing.

## Data Availability

The summary data supporting the reported hydrothermal-aging and hydrocarbon-medium results are included in the article. Individual triplicate values supporting the thermal-oxidative aging results are included in Table 1 as mean ± SD and are available from the corresponding author upon reasonable request. Complete replicate records for the hydrothermal-aging and hydrocarbon-medium datasets were not available for inferential statistical analysis; accordingly, these datasets are reported as descriptive Communication data.
